# Illegal reintroductions of lynx are irresponsible and the wrong path forward for rewilding in Scotland

**DOI:** 10.1007/s13280-025-02146-4

**Published:** 2025-02-21

**Authors:** Toryn Whitehead

**Affiliations:** Department of Geography, King’s College London, 40 Aldwych, London, WC2B 4BG UK

## Abstract

In January 2025 four Eurasian lynx (*Lynx lynx*) were illegally released into the Cairngorms National Park in the Scottish Highlands. All four animals were subsequently captured but unfortunately one lynx died overnight. While the conservation community has widely condemned the unknown perpetrators, possibly guerrilla rewilders, little attention has been given to *why* illegal releases are damaging. Here I outline the three main social-cultural reasons as to why an illegal reintroduction is irresponsible - 1) a breakdown in dialogue and trust; 2) the negative implications for conflict and coexistence; and 3) the spread of misinformation and politicisation – and the disproportionate benefits of a legal reintroduction. Scotland’s history is already littered with illicit releases which have damaged trust and sparked human-wildlife conflicts which otherwise may have been avoided or mitigated. Without patience, empathy, and dialogue to co-produce a reintroduction plan and coexistence strategies, any legal reintroduction of lynx is doomed to fail – let alone an illegal release.

## Introduction

Scotland is suffering from an ecological crisis. One in nine species are at risk of becoming nationally extinct on top of those which have been historically extirpated since the Late Pleistocene (Walton et al. 2023). One response to this has been conservation translocations, often as a part of a broader rewilding movement, which involve the deliberate movement of organisms from one site for release in another (IUCN/SSC, 2013). Reintroductions, where a species is intentionally released inside its native range from which it has disappeared, can be powerful interventions to resurrect ecological functions, helping to restore habitats and ecosystems, when used proactively and creatively (Gaywood et al. 2022). This is particularly true in the UK as many species including lynx cannot cross the English Channel. However, the motivations and objectives of those who conduct wildlife releases can vary widely: from guerrilla rewilders and “milk colonialism" – the transportation of lactating animals such as cows and sheep by European settlers for their milk – to hunting lobbies and eccentric landowners – who, for example, reintroduced the capercaillie (*Tetrao urogallus*) to Scotland in 1837 (Stevenson 2007; Cohen 2020; Thomas 2022). Furthermore, any reintroduction is a time-consuming, expensive, high-risk endeavour – especially if the threats that led to the loss of the species have not been eliminated, or at least reduced to a manageable extent (Gaywood et al. 2022).

Scotland’s history is littered with illicit and contentious reintroductions, the most infamous of which is perhaps the Eurasian beaver. Beavers are ecosystem engineers that have a profoundly positive ecological impact through tree felling and dam building (Stringer and Gaywood 2016). Yet, despite a clear scientific consensus of the ecological benefits, the legal return of beavers had stuttered and faltered for a long time. Frustration at government inaction in part led to the illegal release of beavers into the River Tay catchment by guerrilla rewilders. The illegal reintroduction produced the largest free-living beaver populations in the UK and served to burst the political dam – forcing the Scottish government to grapple with their presence (Thomas 2022). These illicit actions paved the way for numerous legal reintroductions across the UK and so arguably, the beaver bombing of the River Tay catchment has been a rewilding success in many respects. However, there are many social and cultural repercussions of the illegal reintroduction of beavers – and of other species like wild boar (or feral pigs) – such as damage to farmland and illegal and legal persecution (Holmes et al. 2024), which help to demonstrate why an illegal reintroduction of lynx would be irresponsible. Two obvious and important reasons why an illegal reintroduction would be irresponsible is: (1) the potential biosecurity implications of illicit releases – African swine fever in wild boar and tapeworm in beavers, for example – and (2) the welfare implications for the animals themselves who may be unprepared and/or unsuitable for wild release. These are well documented though, so instead I have decided to focus on three important social–cultural reasons as to illustrate the disproportionate benefits of a legal reintroduction, and why an illegal reintroduction is the wrong path forward for lynx in Scotland: (1) a breakdown in dialogue and trust; (2) the negative implications for conflict and coexistence; and (3) the spread of misinformation and politicisation.

## Dialogue and trust

The illegal reintroductions of beavers in Scotland has damaged trust and led to a breakdown in dialogue between some conservation and land management communities (Ward and Prior 2020; Holmes et al. 2024). This is significant because trust and dialogue between actors is foundational for cohesive and effective landscape management to achieve conservation objectives (Redpath et al. 2015; Staddon 2021). This is particularly true for a large carnivore reintroduction, where livestock producers and other rural actors would be required to take on additional burdens from human–lynx coexistence.

Linked to the perceived legitimacy of the reintroduced animal, distrust and dissatisfaction could manifest through illegal killings of lynx – a common causes of reintroduction failure for lynx in Europe (Linnell et al. 2009). Poaching is a common cause of mortality for large carnivores across Europe, and the motivation for such behaviour can be grounded in unfounded rumours of illegal releases by “corrupt" NGOs and perceptions of artificiality, grounded in captivity and genetic hybridisation (Theodorakea and von Essen 2016). The link between legitimacy and persecution is also evident in Scotland, where the persecution of beavers since their illegal reintroduction in the River Tay catchment has been substantial and has persisted despite the introduction of native species status and wildlife legislation protecting them (Holmes et al. 2024). The size of the Scottish beaver population and the legal translocation of “problem" beavers has meant that, so far, the population remains healthy – although this discounts the suffering of individual beavers. A small population of illegally (or legally) reintroduced lynx would be unlikely to withstand these pressures. Therefore, an empathetic and inclusive *legal* process could be critical for minimising the risk of illegal killings and squashing any potential rise of feral politics linked to perceived genetic impurity. The foundation of this process is trust, a currency that itself is rare in Scotland (Ainsworth et al. 2020). But without trust and dialogue, any reintroduction of lynx – legal or illegal – will undoubtably fail.

## Conflict and coexistence

The reintroduction of lynx and their subsequent coexistence with people is complex and will be characterised by opportunities and risks. Conceptualisations of coexistence vary widely across regions and between different species and social groups (Glikman et al. 2021). Broadly though, coexistence is understood to be a state where “people and wildlife can both survive and flourish in their shared environments” (Gao and Clark 2023, p. 2). The most controversial form of coexistence in Scotland is likely to occur between lynx and sheep producers. Norway, which is often used as a point of reference for Scotland, has the highest rate of sheep consumption by lynx (Khorozyan and Heurich 2023). This is likely due to the fact that sheep are free ranging without attendance by shepherds or livestock guardian dogs, making them easier targets for lynx (Odden et al. 2006). The lack of wild prey availability also appears to be an important factor though, as the probability of lynx predating sheep decreases as roe deer density increases (Odden et al. 2013). Despite the fact that wild prey availability would be much higher in Scotland than in Norway (Pepper et al. 2019), livestock producers are still likely to be vulnerable to conflict with lynx. The legal process of reintroduction is critical for reducing this vulnerability – with conservationists and land managers co-producing a shared vision of coexistence and co-developing management strategies for different scenarios. Importantly, this process will not eliminate all conflicts, and negative interactions will still occur, even in a favourable state of coexistence, but these should and can be proactively and effectively managed.

Another significant challenge posed by the reintroduction of lynx in Scotland is the loss of knowledge about lynx and experience of coexistence owing to their long absence from Scottish landscapes. For example, Scottish livestock grazing systems developed in the absence of lynx and other large carnivores, and so no longer implement practices which are designed to prevent livestock predation. This has important implications for coexistence which are well illustrated in Scandinavia. In Norway, the number of livestock killed each year per lynx is 16 (Blanco and Sundseth 2023). Whereas in Sweden, which has a larger population of lynx and, critically, more prevention measures, the number of livestock killed each year per lynx is 0.1 (Blanco and Sundseth 2023). While these measures could be implemented retroactively in response to an illegal reintroduction in Scotland, the process would likely be hostile, arduous and at the expense of multiple human-lynx conflicts with welfare implications for people and lynx.

In the event that effective livestock protection measures were implemented, some sheep predation would still likely occur. Subsequently, it is important to consider the tolerance of livestock producers to predation events. According to the National Sheep Association, any lynx predation of sheep would be “unacceptable” (National Sheep Association 2016, p. 4). This implies that without a fully resourced compensation scheme and preventative measures, human–lynx coexistence is likely to be highly conflictual. Even then, compensation schemes may be inadequate as they would not address some stakeholder’s concerns of psychological distress from lynx predation – a problem reported by Norwegian farmers (Bavin et al., 2023; Zahl-Thanem et al. 2020). Therefore, it is evident that a patient, empathetic dialogue between conservationists and land managers in Scotland would be required to build support for the reintroduction and co-develop a coexistence strategy for the benefit of people and lynx.

## Misinformation and politicisation

Misinformation has real world consequences for conservation, particularly around contentious issues such as large carnivores (Nanni et al. 2020). One potential issue raised by the illegal release of lynx into the Cairngorms was concerns about human safety. Some traditional media outlets used pictures of lynx which emphasised their carnivorous attributes and capitalised on warnings from officials that people should not approach the animals, with headlines such as “‘*Do not approach’: time to get real about the wandering lynx*” (Smith 2025). This is implies that lynx are a threat to people, and unsurprisingly some social media commentary went further, despite there being no reports of fatal lynx attacks in Europe. This fact has not stopped a wariness of lynx manifesting in places where they are extant in Europe. Lescureux et al. (2011) note that negative perceptions of lynx were often based on a lack of experiential and/or academic knowledge. This effect could be stronger in Scotland due to the extinction of experience. Although there are few direct human–lynx interactions due to the animal’s shy and illusive nature, the knowledge acquired from simply sharing the landscape and the direct interactions that do occur could be potentially invaluable. These experiences (or lack of) in places where lynx are extant mean that concerns for human safety are relatively muted. Consequently, in Scotland, the absence of experience-based knowledge may allow myths and wishful thinking about lynx to develop and expand uninhibited. A legal reintroduction would be significantly better placed to quell misinformation through persistent engagement with the general public and key stakeholders – premised on trust and dialogue – before, during, and after the reintroduction. Whereas misinformation about lynx is more likely to become predominant after an illegal release and could have severe implications politically and for human–lynx coexistence, potentially altering human responses to encounters and conflicts.

The illegal release of lynx in the Cairngorms was raised in Scottish parliament on 8th January 2025, leading to condemnation by Scotland’s First Minister John Swinney. Political conversations about lynx in this context are only likely to add to the log jam by generating further political barriers to any future legal reintroduction. Political tensions over wolf conservation in Europe have reached their apotheosis with the potential revision of the Habitats Directive (Ostermann-Miyashita et al. 2025). This politicisation of wildlife is counterproductive, and in Spain and Germany, it has posed a major barrier to collaborative efforts to improve human–wolf coexistence policy (Pettersson et al. 2023; Niedziałkowski 2023). A such, the politicisation of lynx, symbolic of rewilding in Scotland, could serve to damage any legal reintroduction efforts and perhaps even the overall objectives of rewilding in a country where the default on many issues related to conservation is already distrust and polarisation (Redpath et al. 2015).

## Conclusion

The illegal release of lynx was irresponsible, and I have argued that it is the wrong path forward for anyone wishing to see a healthy population of Scottish lynx in the future. It has almost certainly damaged trust and strained relationships between key stakeholders in the conservation and land manager communities, as well as further politicise species reintroductions and lynx in Scotland. The illegal release of lynx has also likely reduced the probability of a legal reintroduction in the near future and may have knock on effects for other conservation activities such as wildcat translocations or deer management. Frustration within the conservation community at official reintroduction processes and regulations which are perceived by some as slow, bureaucratic and risk averse is understandable. Especially when you consider the hypocrisy that the UK has been a vocal supporter of large carnivore initiatives across Europe, without having their own or showing any willingness to meaningfully consider their reintroduction. However, at this critical juncture for nature in Scotland there needs to be less conflict, not more. This incident must spark renewed efforts to build meaningful collaborations between conservationists, land managers and other important actors premised on trust, transparency, and inclusiveness. While this process may take longer, it has the potential to produce ecologically ambitious and socially just outcomes for people and nature. These principles, enacted through official reintroduction processes, are the right path forward for the return of lynx to Scotland too – and have the highest chances of success.
